# Human Breast Tumor Cells Are More Resistant to Cardiac Glycoside Toxicity Than Non-Tumorigenic Breast Cells

**DOI:** 10.1371/journal.pone.0084306

**Published:** 2013-12-13

**Authors:** Rebecca J. Clifford, Jack H. Kaplan

**Affiliations:** Department of Biochemistry and Molecular Genetics, University of Illinois College of Medicine, Chicago, Illinois, United States of America; Wayne State University School of Medicine, United States of America

## Abstract

Cardiotonic steroids (CTS), specific inhibitors of Na,K-ATPase activity, have been widely used for treating cardiac insufficiency. Recent studies suggest that low levels of endogenous CTS do not inhibit Na,K-ATPase activity but play a role in regulating blood pressure, inducing cellular kinase activity, and promoting cell viability. Higher CTS concentrations inhibit Na,K-ATPase activity and can induce reactive oxygen species, growth arrest, and cell death. CTS are being considered as potential novel therapies in cancer treatment, as they have been shown to limit tumor cell growth. However, there is a lack of information on the relative toxicity of tumor cells and comparable non-tumor cells. We have investigated the effects of CTS compounds, ouabain, digitoxin, and bufalin, on cell growth and survival in cell lines exhibiting the full spectrum of non-cancerous to malignant phenotypes. We show that CTS inhibit membrane Na,K-ATPase activity equally well in all cell lines tested regardless of metastatic potential. In contrast, the cellular responses to the drugs are different in non-tumor and tumor cells. Ouabain causes greater inhibition of proliferation and more extensive apoptosis in non-tumor breast cells compared to malignant or oncogene-transfected cells. In tumor cells, the effects of ouabain are accompanied by activation of anti-apoptotic ERK1/2. However, ERK1/2 or Src inhibition does not sensitize tumor cells to CTS cytotoxicity, suggesting that other mechanisms provide protection to the tumor cells. Reduced CTS-sensitivity in breast tumor cells compared to non-tumor cells indicates that CTS are not good candidates as cancer therapies.

## Introduction

Cardiotonic steroids (CTS) are a class of chemical compounds known to specifically inhibit Na,K-ATPase (sodium pump) activity [1], which is responsible for the coupled active transport of Na^+^ and K^+^ ions [2,3] in all human cells. CTS were originally identified in plants and toad venom, and structurally similar compounds have been found endogenously at low levels in mammals. A review by Dvela et al [4] discusses CTS compounds found endogenously in humans, which include the cardenolides; ouabain and digoxin, as well as the bufadienolides; marinobufagenin, 19-nor bufalin, 3b-hydroxy 14a 20:21-bufenolide, Proscillaridin A, and telocinobufagin. The effects that CTS have on cells vary depending on species, Na,K-ATPase isoforms expressed, and the type and dosage of CTS compound used [4]. The CTS compounds used in the present work inhibit the ion pumping function of sodium pump enzymes in human cells by binding to the extracellular surface of the α-subunit of the Na, K-ATPase and confining it to the E2P conformation [5]. When sodium pump activity is inhibited by CTS, intracellular Na^+^ levels increase and reduce the driving force of the Na^+^/Ca^2+^-exchanger to extrude Ca^2+^ from the cells. Ca^2+^ accumulation caused by Na,K-ATPase inhibition increases muscle contractility, making CTS a valuable therapeutic tool in treatment of heart disease [6]. 

In addition to their inhibitory action on Na,K-ATPase, CTS can cause a variety of concentration-dependent cellular responses in epithelial cells. At high CTS concentrations, inhibition of Na,K-ATPase and subsequent Ca^2+^ accumulation can increase reactive oxygen species (ROS), modulate endocytic membrane protein recycling, decrease ATP production, induce growth arrest, and cause cell death [7-10]. Cellular Ca^2+^ accumulation during CTS treatment, enhanced cellular Ca^2+^ entry, and/or internal Ca^2+^ storage release can activate MAPK and Akt signaling pathways [11]. Nanomolar concentrations of CTS have minimal effects on Na,K-ATPase inhibition but can reduce p53 synthesis, and activate signal transduction pathways involving Src, EGFR, Akt, and MAPK [12-16]. Activation of these signaling pathways typically results in increased proliferation and resistance to apoptosis-inducing reagents [17-19]. Drugs capable of inhibiting these signaling cascades are currently being considered as cancer therapies for reducing tumor growth and proliferation [20]. 

CTS compounds have become prospective drugs for cancer treatment, although there is mixed evidence for their effectiveness in reducing incidence and tumor aggressiveness. In 1979, a small cohort study of women with breast cancer demonstrated reduced distant tumor development in women taking digitalis (2 of 33 patients, 6%) compared to women not taking digitalis (28 of 146 patients, 19%) [21]. The 22 year follow-up of those patients showed a death rate of 6% (2 of 32 patients) for digitalis-users compared to 34% (48 of 143 patients) for non-users [22]. Digoxin use has also been described in preventing prostate cancer by as much as 25% according to a large prospective cohort study [23], and an inverse correlation between plasma digitoxin levels and lymphoma/leukemia and kidney cancers has been reported [24]. However, this latter study also found an increased risk of cancer in patients with heart disease regardless of digitalis treatment, which complicates the interpretation of digitoxin effects on cancer incidence in those populations [24]. Other reports fail to provide support for use of CTS as anti-cancer drugs citing either no added benefit or a positive correlation between CTS use and cancer incidence [25-27]. A large cohort study of 143,594 patients observed an increased risk of colon, lung, and prostate cancer in digitalis users [28], and recent studies found digoxin use increased the risk of developing breast cancer in men [29,30] and in post-menopausal women [31]. The odds ratio of breast cancer ranging from 1.25 to 1.39 was congruent with duration of digoxin use [31]. In spite of these mixed outcomes, the potential use of CTS in cancer therapy continues to be suggested. 

To investigate the selectivity of CTS effects on cancer cells, we use several cell lines that have been used extensively for the study of human breast cancer progression. ‘Normal’ non-cancerous cell lines include human mammary epithelial cells (HMEC) 184D and 184A1, obtained from reduction mammoplasty [32], and MCF10A cells. The MCF10 cell series represent stages of cancer progression from non-tumorous (MCF10A) to metastatic invasive ductal carcinoma (MCF10CA1) in a system in which cell lines share a common genetic background [33,34]. In addition to the isogenic estrogen receptor (ER)-negative MCF10A and MCF10CA1 cell lines, we used non-invasive ER-positive MCF7 and invasive ER-negative MDA-MB-231 cells for comparison. The cardenolide, ouabain, is a suggested anti-cancer agent and was used for the majority of this work. Other CTS compounds, bufalin and digitoxin, are also prospective cancer therapeutic drugs and used in this work to compare their ability to inhibit the enzymatic function of the sodium pump with their impact on cell viability. We demonstrate that the effects of CTS on Na,K-ATPase activity, cell proliferation, kinase activation, and viability in cells with varying metastatic potential indicates that malignant breast cells are no more sensitive to CTS cytotoxicity than are non-tumorigenic cells.

## Materials and Methods

### Cell Culture

MCF10A, MCF7, and MDA-MB-231 were obtained from ATCC (Manassas, VA). The MCF10CA1 and MCF10AT immortalized cell lines were gifted by Dr. Fred Miller (Barbara Ann Karmanos Cancer Institute, Detroit, MI,) and created by introducing a T24 Ha-ras oncogene into MCF10A cells, xenografting transformed cells into mice, and selecting advanced lesions [33,35]. HMEC pre-stasis 184 (Batch D) and immortalized 184A1 cells were obtained from Dr. Martha Stampfer (Lawrence Berkeley National Laboratory, Berkeley, CA). A2780 (cisplatin sensitive) and A2780CP (cisplatin resistant) ovarian cancer cell lines were a kind gift from Dr. T.C. Hamilton (Fox Chase Cancer Center, Philadelphia, PA) [36]. All cell lines were maintained in a humidified incubator with 5% CO_2_ at 37°C. Growth medium containing Dulbecco's Modified Eagle's Medium (Mediatech, Manassas, VA) supplemented with 25 mM HEPES buffer (Mediatech, Manassas, VA) and 10% fetal bovine serum (Atlanta Biologicals, Lawrenceville, GA) was used for MCF7, MDA-MB-231, and A2780/CP cell lines. MCF10 series cell lines were grown in Dulbecco's Mmodified Eagle's Medium/Ham's F-12 Nutrient Mixture 1:1 growth medium supplemented with 5% horse serum, 25 mM HEPES, 20 ng/ml epidermal growth factor, 10 ng/ml cholera toxin, 10 µg/ml insulin, and 500 ng/ml hydrocortisone. HMEC cells were grown in M87A medium supplemented with 0.5 ng/ml cholera toxin and 0.1 nM oxytocin [32]. 

### Membrane Preparation

Total membranes (TM) were prepared from cells as previously described [37] and resuspended in cold homogenizing buffer (10 mM Tris-HCl, 2 mM EDTA, 250 mM sucrose, pH 7.4) containing Complete-Mini Protease Inhibitor Cocktail Tablets (Roche, Indianapolis, IN). Total cell lysate preparations were made by incubating cell pellets in lysis buffer (150 mM NaCl, 50 mM Tris, 1% CHAPS, pH 7.4) for 30 minutes at 4°C. Lysates were cleared at 15,000 x g for 10 minutes and the soluble protein supernatant was collected for analysis. Protein concentrations were determined using the Coomassie Protein Assay Kit (Pierce, Rockford, IL). 

### Na,K-ATPase Activity Assay of Total Membranes

The ATPase activity assay of isolated TM from cells was performed for 30 min at 37°C as previously described [38]. Where appropriate, various concentrations of FBS, ouabain, digitoxin, or bufalin were added to TM samples at the same time as ATP and concentrations were maintained for the duration of the assay. Na,K-ATPase activity was determined as μmol of phosphate liberated per milligram of protein per hour, and displayed as percent of activity relative to untreated samples. 

### Cell Growth and Viability

To determine cell growth curves, cells were trypsinized, counted using a Scepter Cell Counter (Millipore, Billerica, MA), plated in a 96-well plate at a density of 1,000, 3,000, 5,000, or 10,000 cells per well, and allowed to attach overnight. On day 2, growth curves were determined using the PrestoBlue Viability Assay (Life Technologies, Grand Island, NY) to ensure cell densities used for viability and proliferation assays were in linear growth phase. Viability assays were performed by plating 3 x 10^3^ cells per well in 96 well plates, and grown overnight to approximately 80% confluence prior to addition of fresh growth media with or without serum, ouabain, 5 µM extracellular signal-regulated kinase (ERK) inhibitor 6-(4-bromo-2-chloroanilino)-7-fluoro-N-(2-hydroxyethoxy)-3-methylbenzimidazole-5-carboxamide (AZD6244; Selleck Chemicals, Houston, TX), and/or 10 µM Src family kinase inhibitor 4-Amino-5-(4-chlorophenyl)-7-(t-butyl)pyrazolo[3,4-d]pyrimidine (PP2; Millipore, Billerica, MA). Cell viability was assessed using the PrestoBlue Viability Reagent and displayed as the percentage of living cells from treated samples relative to untreated control samples. 

### Cell Proliferation

Cells plated at 3 x 10^3^ cells per well were grown to 80% confluence in 96 well plates and treated with ouabain for two or 24 hours prior to Bromodeoxyuridine (BrdU) Cell Proliferation Assay (Calbiochem, Billerica, MA). BrdU label was added to cells in fresh culture medium with or without ouabain for two hours according to manufacturer’s recommendations. BrdU incorporation into the DNA of proliferating cells was detected using an anti-BrdU primary antibody and Peroxidase Goat Anti-mouse secondary antibody. Fluorescence was quantified using a FLUOStar Omega microplate reader (BMG Labtech). 

### Apoptosis Assay

MCF10A, MCF10CA1, MCF7, or MDA-MB-231 cells were grown on glass slides for 48 hours and treated with or without 500 nM ouabain or 50 nM bufalin for 6 hours. As a positive control, MCF10A and MCF10CA1 cells were treated with DNaseI for 15 minutes prior to assay. Cells were fixed and permeabilized prior to Terminal deoxynucleotidyl transferase dUTP nick end labeling (TUNEL) staining of DNA fragmentation using the ApopTag Fluorescein In Situ Apoptosis Detection Kit according to manufacturer’s instructions (Millipore, Billerica, MA). Slides were covered with Vectashield Mounting Medium with DAPI (Vector Laboratories, Burlingame, CA) and sealed with a glass coverslip. Images were detected and analyzed using Zeiss LSM 5 Pascal microscope and software. 

### Western Blot Analysis

Protein samples were subject to western blot analysis according to previously described procedures [39]. Transferred proteins were blocked using either 5% dried milk, 0.1% Tween-20 in phosphate-buffered saline or 5% bovine serum albumin, 0.1% Tween-20 in Tris-buffered saline. PVDF membranes were sequentially probed with primary antibodies and horseradish peroxidase-conjugated secondary antibodies according to manufacturer’s recommendations. Luminata Forte Western HRP Substrate (Millipore, Billerica, MA) was used for peroxidase detection and the resulting signal intensity was quantified on a BioRad Chemidoc XRS using Quantity One Version 4.6.2 software. When appropriate, signal intensity from anti-actin antibody was used as a protein loading control.

### Immunoprecipitation

TM from MCF10A and MDA-MB-231 cells was collected and 50 µg of protein was subject to immunoprecipitation (IP). Protein was solubilized in IP buffer (150 mM NaCl, 50 mM Tris-HCl, and 1% CHAPS, pH 7.5), and centrifuged at 20,000 x g for 10 minutes. 10% of the solubilized protein in the supernatant was used as input for western blot. The remaining supernatant was subject to IP by incubating for 24 hours at 4°C with rabbit anti-α M4/M5 antibody and 30 µl Immobilized Protein A/G-agarose beads (Thermo Fisher, Rockford, IL). 10% of the supernatant was collected and used as unbound protein. The IP beads were washed three times for 5 min each with IP buffer, twice with high salt wash buffer (0.5 M NaCl, 50 mM Tris-HCl, 5 mM EDTA, pH 7.4), and twice with low salt buffer (10 mM Tris-HCl, pH 7.4) to remove unassociated proteins. Followed each wash, samples were spun by centrifugation at 500 x g for 1 minute, and supernatant was removed by aspiration. Immunoprecipitated proteins were eluted from the beads with 2x Laemmli Sample Buffer containing 5% 2-mercaptoethanol and resolved by western blot analysis.

### Antibodies

Rabbit anti-α M4/M5 antibody was used for IP at a 1:200 dilution as previously described [37]. Antibody dilutions used for immunoblotting were as follows: 1:10,000 of mouse Na,K-ATPase anti-α_1_ or Na,K-ATPase β_1_-subunit (Affinity Bioreagents, Golden, CO), 1:1000 mouse anti-actin JLA20 (Developmental Studies Hybridoma Bank, Iowa City, IA), 1:1000 of either rabbit anti-total ERK1/2 or mouse anti-phospho-ERK1/2 (Santa Cruz, Dallas, TX), 1:1000 of mouse anti-p53 (Santa Cruz, Dallas, TX), 1:500 mouse anti-Src (Millipore, Billerica, MA). Peroxidase AffiniPure goat anti-mouse or goat anti-rabbit (Jackson Immunoresearch, West Grove, PA) secondary antibodies were diluted 1:5000. 

### Statistical Significance

Data are expressed as mean ± standard error (SE) and (n) values represent the number of independent experiments performed. In some experiments, data was calculated as a percentage of the untreated control sample mean. Each experiment was performed at least in triplicate. P values were calculated utilizing Student's t-test or by one-way ANOVA. A P value <0.05 was considered the level of statistical significance.

## Results

### Cell Lines

In order to test the hypothesis that CTS are appropriate potential therapies for cancer it was necessary to investigate whether or not the reported cytotoxicity of CTS showed selectivity for cancer cells over non-tumorigenic cells. Primary pre-stasis 184D and immortalized 184A1 cells are non-cancerous human mammary epithelia cells. MCF10A cells are immortalized cells frequently used in breast cancer studies as a ‘normal’ control due to their inability to form tumors in mouse xenografts [35]. While they were derived from the mammary gland of a female patient with fibrocystic disease, they are non-tumorigenic, and display morphology typical of mammary epithelial cells [40]. MCF10CA1 cells are fully malignant resulting in undifferentiated to differentiated carcinomas in xenografts [33-35]. In addition, we compared CTS responses in ER-positive MCF7 and ER-negative MDA-MB-231 cells, as they are frequently used for comparing aspects of breast cell tumorgenicity and represent characteristics commonly found in breast cancer in vivo [41].

### Na,K-ATPase Inhibition by Cardiac Glycosides

**Figure 1:**
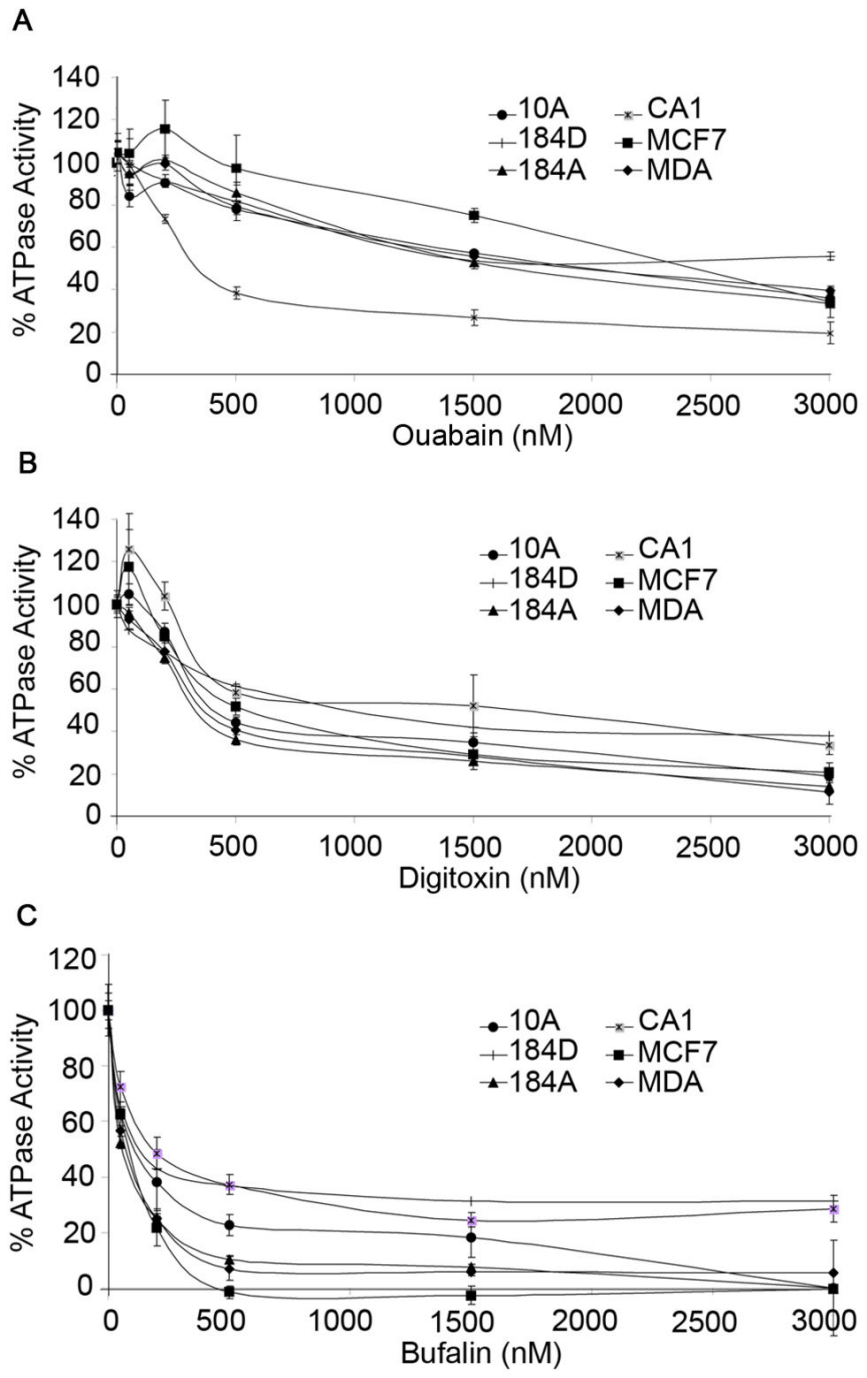
ATPase Assay of breast cell membranes treated with cardiac glycosides; (A) ouabain, (B) digitoxin, and (C) bufalin. The Na,K-ATPase activity in total membranes isolated from non-tumorous 184D, 184A, and MCF10A (10A) cells, as well as cancerous MDA-MB-231 (MDA), MCF7, and MCF10CA1 (CA1) cells is inhibited in a concentration dependent manner for all cells types. Na,K-ATPase activity (µmol Pi liberated per mg protein per hour) was determined and displayed as the percentage of activity relative to the untreated sample for each cell type. There was no statistically significant difference in activity between normal and tumor cells treated with similar concentrations of ouabain, digitoxin, or bufalin (p>0.05).

To assess the effects of CTS compounds on Na,K-ATPase activity, TM was collected (see Methods) from non-tumorigenic MCF10A, 184D, 184A1, oncogenic MCF10CA1, non-invasive MCF7, and invasive MDA-MB-231 breast cells, and treated with various concentrations of ouabain, digitoxin, or bufalin (Figure 1A-C). The assay revealed that CTS compounds inhibit Na,K-ATPase activity in a concentration-dependent manner. Bufalin is most effective at reducing activity at low concentrations, whereas digitoxin and ouabain required higher concentrations for more complete Na,K-ATPase inhibition. All cell lines have Na,K-ATPase activity that is significantly inhibited by the CTS compounds regardless of their oncogenic status. Each drug inhibits Na,K-ATPase activity in cancer or non-tumorous cell types with comparable potency, although bufalin is more potent than ouabain or digitoxin (Table 1). 

**Table 1:** ATPase activity inhibition by CTS.

	**Ouabain**	**Digitoxin**	**Bufalin**
MCF10A	2013.65±50.0	1346.66±85.2	475.44±96.4
184D	1185.3±81.3	2068.9±306.6	384.6±36.5
184A1	2461.9±220.2	1827.4±88.7	295.9±10.5
MCF7	1975.1±32.7	1163.4±42.7	183.6±17.0
MDAMB231	2123.9±44.2	1178.2±154.5	169.9±13.3
MCF10CA1	2492.4±266.9	1482.1±73.8	164.6±9.1

IC_50_ values for CTS inhibition of ATPase activity in each cell type were calculated using the four-parameter logistic equation for CTS concentration (nM), and displayed as mean ± SE, n>3.

### Cardiac Glycoside Effects on Cell Proliferation

**Figure 2:**
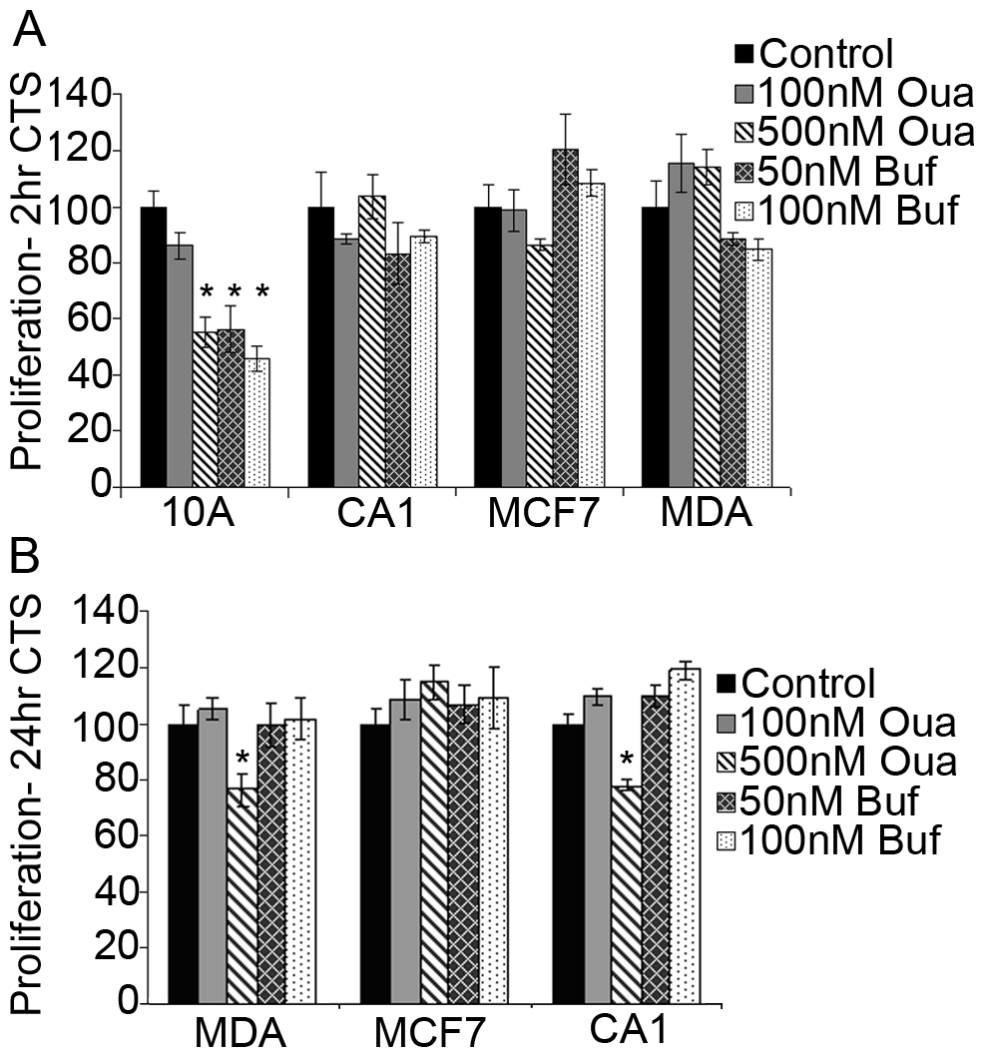
Proliferation of CTS-treated cells. A) Normal MCF10A cells exhibited a concentration-dependent decrease in BrdU incorporation (proliferation) when treated with 500 nM ouabain, 50 nM bufalin or 100 nM bufalin for 2 hours (p<0.01). MCF7, MCF10CA1, and MDA-MB-231 cells showed no significant decrease (p>0.05) in proliferation under comparable conditions. MDA-MB-231 cells treated with 50 nM or 100 nM bufalin had p-values of 0.057 and 0.061, respectively. B) High ouabain treatment (500 nM) for 24 hours caused a decrease in proliferation of MDA-MB-231 and MCF10CA1 cells, but no change to proliferation of MCF7 cells. Low ouabain or bufalin treatment had no effect on proliferation of any cancer cells tested.

Sodium pump activity is a central player in cellular ion homeostasis, volume control, and cell viability. Decreasing its ability to transport Na^+^ and K^+^ ions in and out of the cell causes a series of downstream effects on other ion transporters and signaling molecules that can affect cell differentiation, proliferation, and viability. Since the Na,K-ATPase activity in isolated breast cell membranes from non-tumorigenic and cancer cells showed comparable inhibition by CTS (Figure 1), it was important to determine if the cellular effects of Na,K-ATPase inhibition were also similar . To address this issue we employed a BrdU assay (see Methods) that measures cell proliferation by incorporating bromodeoxyuridine into the DNA of actively dividing cells. Cells were grown to 80% confluency and pre-incubated with ouabain or bufalin for 2 (Panel 2A) or 24 (Panel 2B) hours. BrdU was then added and for 2 hours the amount incorporated into DNA of dividing cells was determined by fluorescence intensity using anti-BrdU antibodies. MCF10A cells (non-tumorigenic) exhibited a decrease in proliferation when treated with 500 nM ouabain, 50 nM or 100 nM bufalin for 2 hours (P<0.001) (Figure 2A). Oncogene-transfected MCF10CA1 and tumor-derived MDA-MB-231 cell proliferation was not affected by short-term ouabain or bufalin treatment (Figure 2A), but 24 hour ouabain at high concentrations did cause a slight decrease in proliferation (Figure 2B). Determination of the proliferation rates of MCF10A cells treated with CTS for 24 hours was not possible, as all of the cells detached after the long-term treatment. In contrast, MCF7 cells showed no decrease in proliferation in the presence of similar concentrations of CTS for either short or long-term incubation. Thus, although ouabain or bufalin cause comparable inhibition of Na,K-ATPase activity (Figure 1 & Table 1), normal cells have reduced proliferation after short-term ouabain or bufalin treatment, whereas tumorigenic cells show little effect. Thus, cancer cells are more resistant to the anti-proliferative effects of CTS than non-tumorigenic cells.

### CTS and Cell Viability

**Figure 3:**
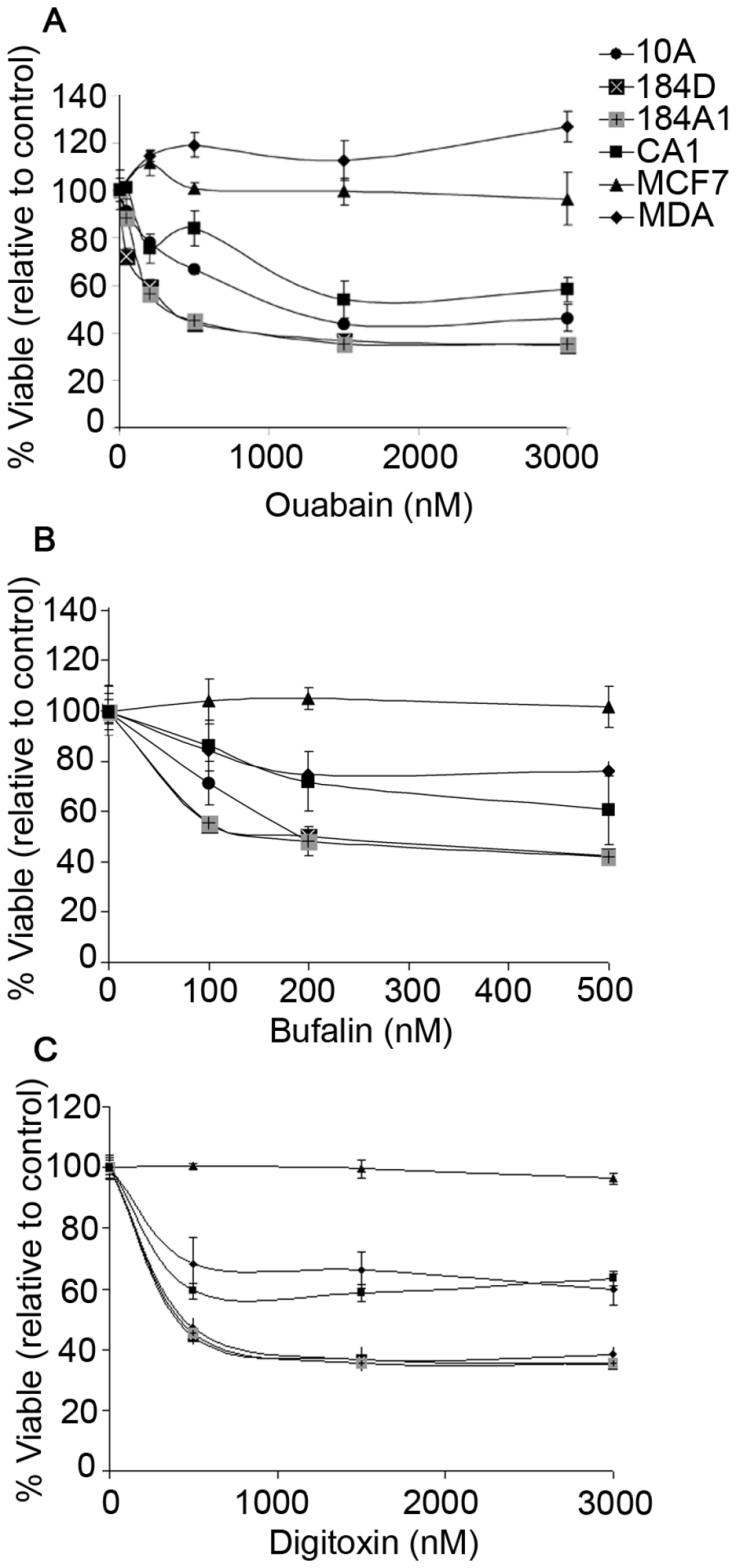
Viability of CTS-treated cells. Normal 184D, 184A1, and MCF10A cells and tumorous MCF10CA1, MCF7, and MDA-MB-231 cells were treated with (A) ouabain (B) bufalin, or (C) digitoxin for 24 hours, and viability was determined by PrestoBlue Viability assay. Normal cells exhibited a concentration-dependent decrease in viability when treated with all CTS compounds tested. Tumor MCF7 and MDA-MB-231 cells were more resistant to CTS-induced cell death than MCF10A cells. Level of significance was determined (p<0.05) using ANOVA for group comparisons or Student’s t-test for individual comparisons.

To further characterize the chronic impact of CTS on breast cells, we treated MCF10A, 184D, 184A1, MCF7, MDA-MB-231, and MCF10CA1 cells for 24 hours with various concentrations of ouabain, digitoxin, or bufalin and measured cell viability (Figure 3A-C) using PrestoBlue Viability assay (See Methods). Cell viability was decreased in 184D, 184A1, and MCF10 series cell lines treated with ouabain and digitoxin concentrations >500 nM and bufalin >100 nM. IC_50_ values are displayed in Table 2. MCF7 cells were unaffected by any concentration of CTS and MDA-MB-231 cells were less resistant to CTS toxicity than any normal cell line at high concentrations. At high concentrations, all cancer cell lines tested were significantly more viable than the normal cells. 

**Table 2:** Reduced cell viability (IC_50_) by CTS.

	**Ouabain**	**Digitoxin**	**Bufalin**
MCF10A	2170.47±192.3	1572.9±191.2	465.2±25.9
184D	1354.18±155.1	1272.2±65.4	329.0±11.2
184A1	1494.1±45.3	1442.7±29.3	318.9±6.1
MCF7	X	X	X
MDAMB231	X	3681.7±149.6	936.4±4.9
MCF10CA1	2667.1±574.1	3682.3±89.5	635.2±171.8

The decrease in cell viability (IC_50_) by CTS was determined for each cell type using the four-parameter logistic equation for CTS concentration (nM), and displayed as mean ± SE (n>3). ‘X’ designates that no IC_50_ value could be calculated.

### CTS Treatment Duration and Serum Concentration Affect Cell Viability

**Figure 4:**
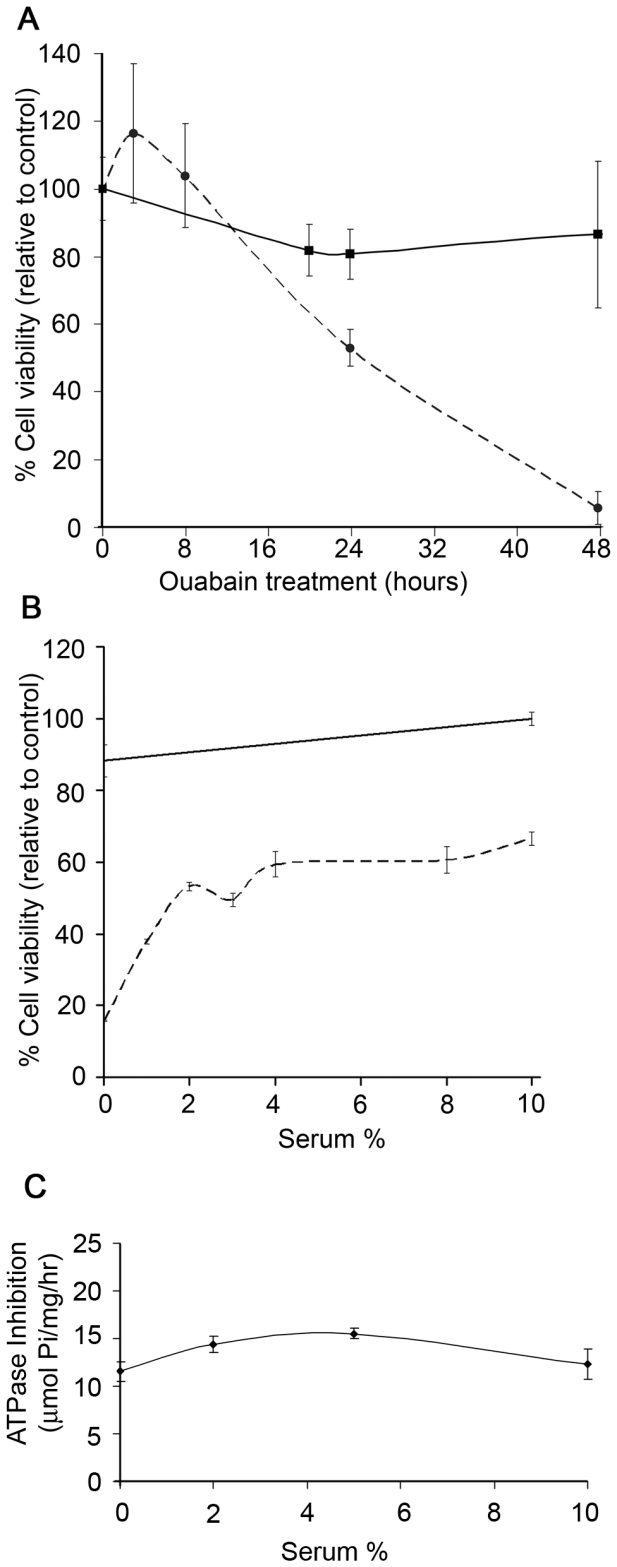
CTS-treated MCF10A cell viability is time-dependent and serum-sensitive. A) MCF10A (dashed line) cells incubated with 500 nM ouabain showed a time-dependent decrease in viability during CTS treatment. MDA-MB-231 (solid line) viability was unaffected during the 48 hour duration of ouabain treatment. B) MCF10A cells grown with serum deprivation or with serum concentrations less than 5% had reduced viability when treated with 500 nM ouabain for 24 hours (dashed line). MDA-MB-231 cell viability was not affected by ouabain treatment when grown in 0-10% serum concentrations (solid line). C) MCF10A isolated membranes were incubated with 0-10% serum with or without 500 nM ouabain prior to ATPase assay. The difference in ATPase activity between untreated and ouabain treated was measured and displayed according to serum level. No significant difference in ATPase inhibition by ouabain exists from altering serum levels (p > 0.05).

In Figure 3, ouabain decreased normal non-tumorigenic cell viability in a concentration-dependent manner. To assess whether the duration of CTS treatment also impacts cell viability, ouabain-sensitive MCF10A and ouabain-resistant MDA-MB-231 cells were incubated with 500 nM ouabain for 0 to 48 hours (Figure 4A). A linear decrease in viability is observed between 8 and 48 hours treatment in MCF10A cells. After 2 days of ouabain treatment, nearly all of the MCF10A cells have died; in contrast MDA-MB-231 cell viability is only slightly affected after similar treatment durations (Figure 4A). 

It has been shown that endogenous ouabain is present in serum and at low levels it can enhance cell proliferation and viability [42]. To determine whether the presence of serum in growth medium affects the response of CTS on cell health, we incubated MCF10A or MDA-MB-231 cells for 24 hours in growth medium containing from 0-10% serum, followed by 24 hours of ouabain treatment at 500 nM (Figure 4B). Reduced levels of serum in the media of MCF10A cells led to increased ouabain sensitivity (Figure 4B, dashed line). It is possible that binding of ouabain by serum enhances the viability of ouabain-treated MCF10A cells (as seen in Figure 4B) by reducing its bioavailability, and thus Na,K-ATPase inhibition. To test this hypothesis, we incubated MF10A cell total membranes in 0-10% serum, with or without 500 nM ouabain, and performed ATPase assays (Figure 4C). We observed that serum does not significantly (P>0.05) alter ouabain’s ability to inhibit Na,K-ATPase enzymes in isolated cell membranes. Thus, ouabain added to cultured cells is not bound by serum in growth media, and it is bioavailable. 

### Apoptosis following Ouabain Treatment

**Figure 5:**
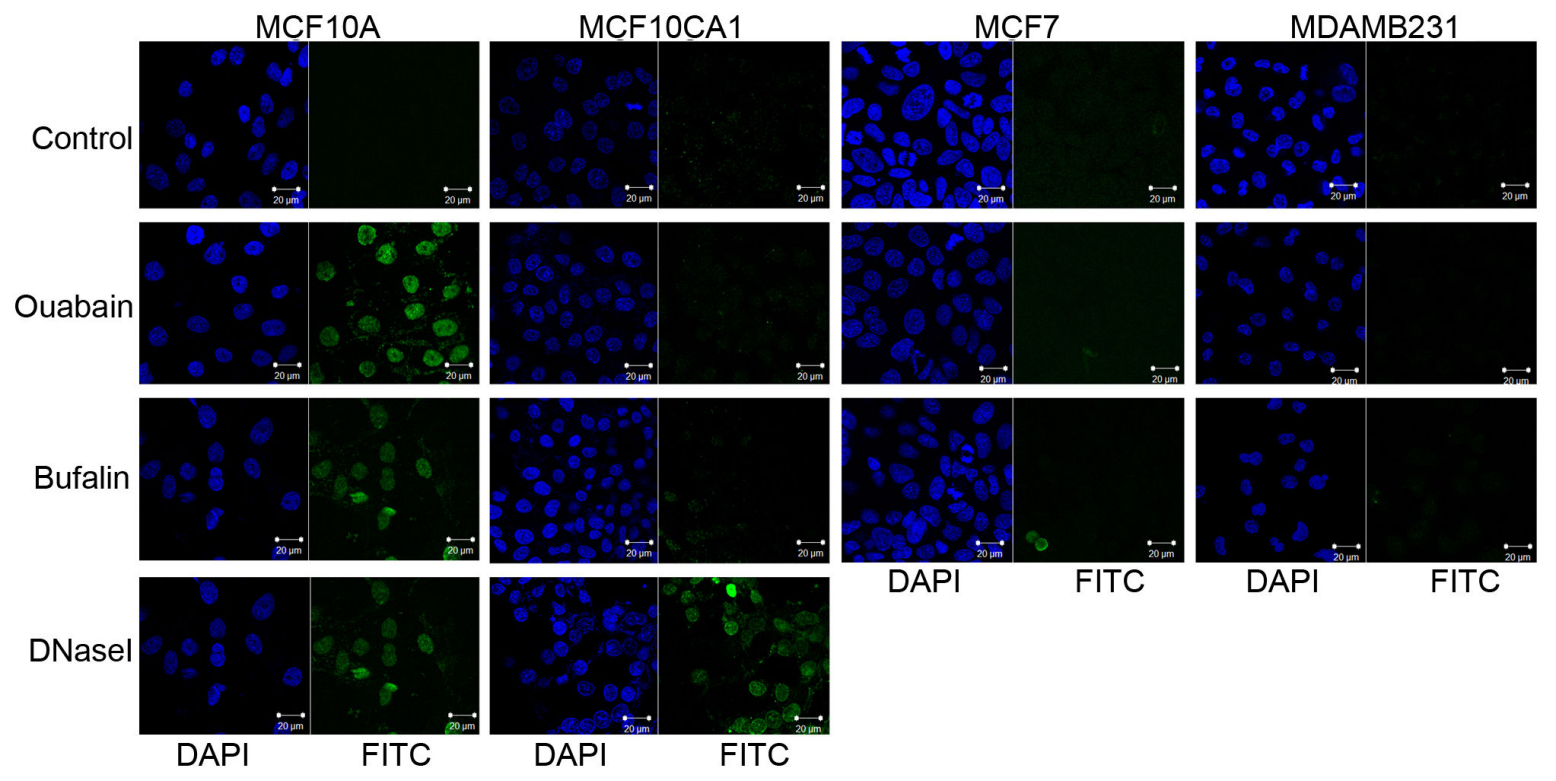
Apoptosis in normal and cancerous cells. Ouabain causes MCF10A cells to undergo apoptosis, but not MCF7, MDA-MB-231, or MCF10CA1 cells. DNA fragmentation (free 3’-OH ends) was visible by FITC (green) staining using the TUNEL assay when MCF10A cells were treated with 500 nM ouabain or 50 nM bufalin for 6 hours. DNaseI was included as a positive control for DNA fragmentation of in MCF10A and MCF10CA1 cells. DAPI (blue) was used to counter-stain the nucleus, and images were collected using Zeiss Confocal Microscope and Imaging Software.

To determine if cells are induced to undergo apoptosis as a consequence of CTS treatment, we utilized the TUNEL assay (see Methods) on cells treated with CTS. The CTS treatment duration of 6 hours was used to visualize DNA fragmentation in cells undergoing apoptosis without causing detachment of dying cells from the substrate. Untreated and DNase-treated cells were included as negative and positive controls, respectively. Over 90% of MCF10A cells incubated with either 500 nM ouabain or 50 nM bufalin exhibited DNA fragmentation, a marker for apoptosis, compared to <5% of tumorigenic MCF10CA1, MCF7, and MDA-MB-231 cells (Figure 5). Thus it appears that the tumor cells are more resistant to the drug-induced apoptosis.

### Signaling Activation following CTS Treatment

**Figure 6:**
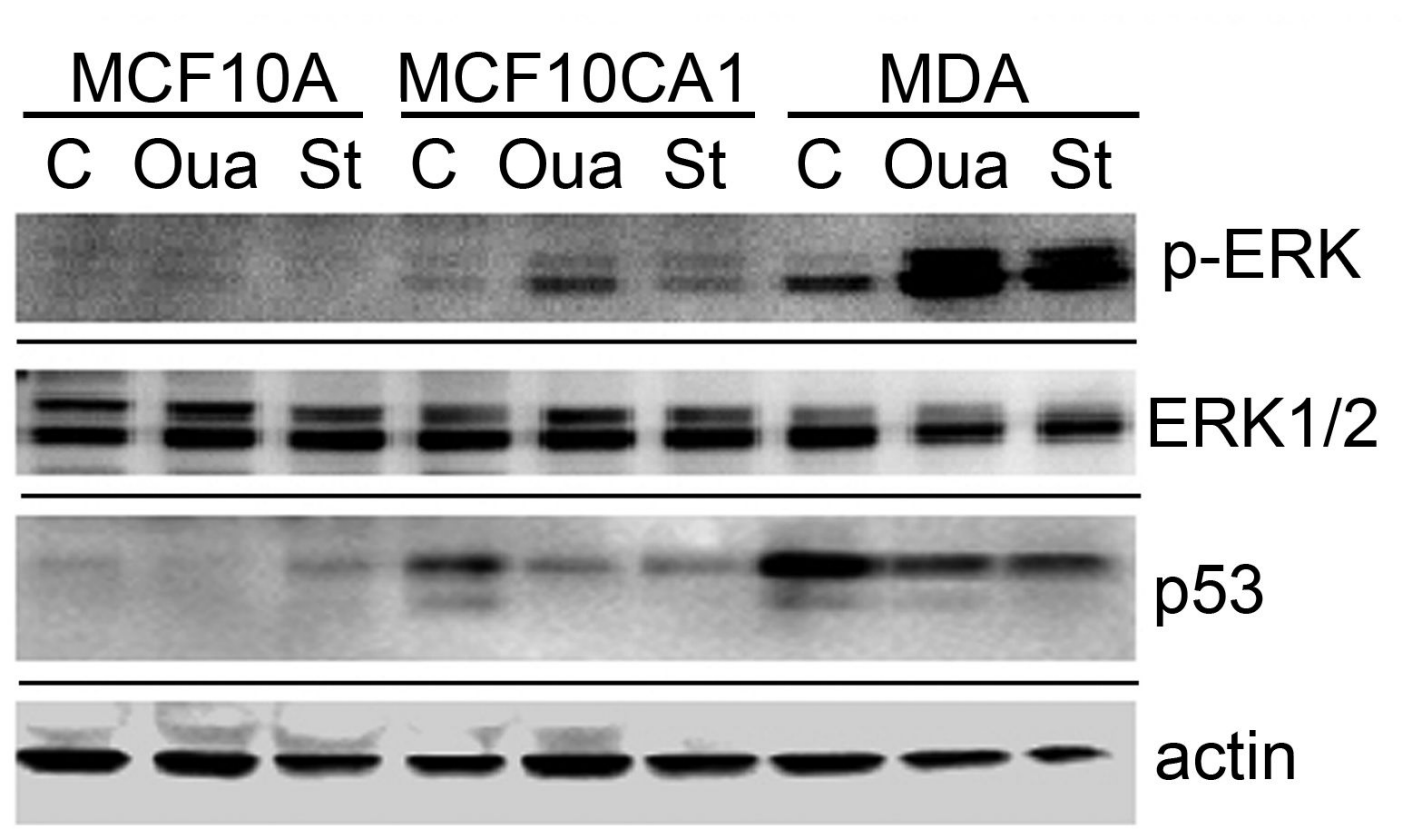
Protein expression in cells with or without CTS treatment. Lysates from MCF10A, MCF10CA1, and MDA-MB-231 cells treated with ouabain (Oua) or staurosporine (St) were analyzed with western blot using anti-actin, p53, total ERK1/2, or p-ERK antibodies. Western blot shows tumor cells contain altered levels of p53, and p-ERK after long-term ouabain treatment.

Previous reports demonstrate ERK1/2 activation occurs in cancer cells in response to short-term (<120 minute) CTS treatment [14,16,43]. The ability of cancer cells to resist apoptosis during long-term CTS treatment (Figures 3 & 4A) led us to consider whether ERK1/2 signaling kinases are activated during long-term CTS treatment, which might enable proliferation and cell survival mechanisms. Western blot analysis was performed using lysates from normal and tumor cells that had been treated (or not) with 500 nM ouabain or stuarosporine (as a positive control) for 24 hours (Figure 6). Untreated cell lysates revealed a significant difference in the level of p53 protein expression from each cell type. A mutated form of p53 is found in over 50% of breast cancer cells and often results in gain-of-function oncogenic activity [44,45]. Mutant p53 is expressed in MDA-MB-231 cells, whereas wild-type p53 is expressed in MCF10CA1 and MCF10A cells. The expression of p53 was decreased in both cancer cells when treated with ouabain, whereas MCF10A cell lysates had only trace levels of p53 under all treatment conditions. Total ERK1/2 protein expression was similar in all untreated cells; however, long-term ouabain treatment elevated p-ERK1/2 levels in lysates from MCF10CA1 and MDA-MB-231 cancer cells (Figure 6). Normal MCF10A cell lysates did not contain detectable levels of activated p-ERK1/2 under any treatment conditions. Thus the high levels of activated p-ERK1/2 seen in tumor cells are further enhanced by long-term CTS treatment. 

### Ouabain Mediated Interaction of Src/Na,K-ATPase

**Figure 7:**
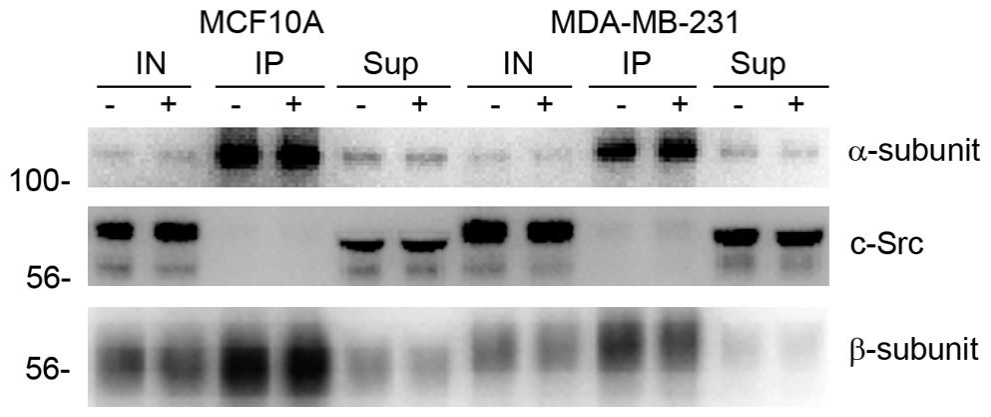
Src kinase does not coimmunoprecipitate with α-subunit in MCF10A or MDA-MB-231 cells. MCF10A and MDA-MB-231 cells were treated with (+) or without (-) 500 nM ouabain for 24 hours. Lysates were collected and subject to immunoprecipitation using a rabbit anti-α antibody, raised against Na,K-ATPase α-subunit’s M4/M5 cytoplasmic loop. 10% of the initial lysate (IN), the co-immunoprecipitated proteins (IP), and 10% of the unbound supernatant (Sup) after IP were used for western blots. Western blot analysis was performed and probed using mouse anti-Na,K-ATPase α_1_-subunit, mouse anti-Src, and mouse anti-Na,K-ATPase β_1_ subunit antibodies.

Previous reports of Src activation in response to ouabain treatment indicate that it may also enable cell survival [46]. It has been proposed that ouabain interaction with Na,K-ATPase directly mediates Src activity and downstream proliferative effects. A recent study suggests that under normal conditions, the Na,K-ATPase α-subunit interacts directly with Src at its SH2 and kinase domains, preventing Src activation and subsequent proliferation and differentiation [47,48]. The Src kinase domain’s interaction with Na,K-ATPase is alleviated by ouabain, thus enabling Src activity. The majority of this previous work was done using either purified isolated proteins or cells overexpressing Src. Another study proposes that ouabain’s activation of Src is not mediated via Na,K-ATPase interactions, but rather via a change in the cellular ATP/ADP ratio after Na,K-ATPase inhibition [49]. Since the activation of Src has multiple downstream effects which regulate cell survival and proliferation, and reported interactions between Src, Na,K-ATPase, and ouabain may mediate those effects, we wanted to determine if endogenous levels of Src interact with the Na,K-ATPase enzyme in the breast cells. To detect if the α-subunit can be found in complex with Src endogenously and if those interactions are affected by ouabain, we immunoprecipitated the Na,K-ATPase α-subunit from total membranes of MCF10A or MDA-MB-231 cells treated with or without 500 nM ouabain for 24 hours (Figure 7). These data were obtained using a highly efficient immunoprecipitating antibody for the Na,K-ATPase [37]. Na,K-ATPase α-subunit was pulled down by the immunoprecipitating anitbody; however, there was no indication of Src kinase in the immunoprecipitated protein complex as the majority of Src was present in the unbound supernatant (Figure 7). Western blots were also probed for Na,K-ATPase β_1_ subunit, a known interaction partner of α-subunit, to ensure that protein complexes remained intact during solubilization treatment (Figure 7). Thus we find no evidence of endogenous Src/Na,K-ATPase complexes in non-tumorous MCF10A or invasive MDA-MB-231 cells. 

### Signaling Inhibition Does Not Increase Ouabain Sensitivity in Cancer Cells

The activation of ERK1/2 (Figure 6) and Src [46] in response to CTS in cancer cells indicates that the Src/Ras/MAPK signaling pathways may play a role in protecting tumor cells from apoptosis. If CTS cause activation of ERK and Src and this activation protects the cells from CTS cytotoxicity, perhaps CTS treatment in the presence of ERK or Src inhibition would render the tumor cells more sensitive to CTS. To test this hypothesis, we incubated the CTS-resistant MDA-MB-231 or MCF7 cells with 500 nM ouabain and either 5 µM ERK inhibitor AZD6244, or 10µM Src inhibitor PP2, for 24 hours and then assayed cell viability (Table 3). Co-treatment of either Erk or Src inhibitors with ouabain did not significantly affect cancer cell viability compared to ouabain treatment alone (p>0.05). Thus, cancer cell resistance to CTS cytotoxicity is not due to elevated ERK or Src activation, suggesting that others mechanisms provide protection to the cells. 

**Table 3:** ERK and Src inhibitors do not sensitize tumor cells to CTS-induced cell death.

**Treatment**	**MCF7**	**MDAMB231**
Control	100 ± 3.04	100 ± 17.7
500 nM Ouabain	89.56 ± 3.58	85.06 ± 12.69
5 µM ERK Inhibitor	104.10 ± 0.26	103.66 ± 10.42
10 µM Src Inhibitor	97.08 ± 2.26	86.60 ± 12.94
Ouabain + ERK Inhibitor	83.69 ± 1.72	77.60 ± 9.51
Ouabain + Src Inhibitor	81.14 ± 2.26	76.82 ± 12.15

Viability is displayed as the percentage of viable cells relative to untreated control; mean ± SE; n=3. Differences in viability from treatments with ouabain only compared to with ouabain plus inhibitor were not statistically significant (p>0.05).

## Discussion

Cardiotonic steroids are digitalis-like drugs that are widely used for treating heart failure. These drugs inhibit Na,K-ATPase activity causing increased intracellular sodium and subsequent calcium elevation. In cardiac myocytes, Ca^2+^ accumulates in the sarcoplasmic reticulum and is released during depolarization resulting in increased muscle contractility [6,50]. The effect that CTS drugs have on muscle contractility has made them valuable therapeutic tools for heart failure and cardiac arrhythmias [51-53]. While beneficial in cardiac insufficiency, Na,K-ATPase inhibition can also induce apoptosis in a variety of cell types. Sensitizing cells to apoptosis is a potentially valuable strategy in the development of new anti-cancer drugs. Several digitalis-like drugs have been proposed as anti-cancer drugs, based on their ability to target tumor cells to apoptotic pathways [54], or due to the inability of cancer cells to confer drug resistance to them, such as the synthetic digitalis-like compound 19-hydroxy-2″oxovoruscharin [55]. Earlier studies observe a range of apoptotic effects and signaling changes in response to CTS in metastatic cells; however, comparisons of CTS effects in cancer cells with normal cells are limited. The *sine qua non* for an effective cancer therapy is the specificity of such actions in tumor cells versus normal cells. In the present work we have systematically compared the responses of cancer cells and normal cells to the actions of CTS molecules. Our results provide evidence that normal cells are highly vulnerable to the actions of CTS in stimulating apoptosis, slowing proliferation, and limiting viability. 

Membranes isolated from normal and cancerous cells contain Na,K-ATPase activities that show comparable sensitivity to inhibition by the cardiac glycosides, ouabain, bufalin, and digitoxin (Figure 1). All human α-subunit isoforms contain a conserved ouabain-binding motif [56], thus it is expected that CTS-sensitivity is similar for all cell lines used in this work. However, ouabain-induced ERK signaling is affected differently by α-isoform expression, with α1, α3 and α4 allowing signal transduction, and α2 having no effect on ERK activation [57]. Furthermore, two plasma membrane pools of Na,K-ATPase α-subunits has been proposed, in which the caveolar fraction contains a non-pumping pool of α-subunits which functions in signal transduction, and the non-caveolar fraction contains a pumping pool which functions in ion homeostasis [58]. This work was later disputed by Liu et al, in which they found that caveolar and non-caveolar fractions contain similar ATPase activities and signaling function, and that the caveolar fraction does not contain a non-pumping pool of Na,K-ATPase [59]. This evidence that both plasma membrane pools of α-subunits are functionally active, implies that if intact cells show differing susceptibility to the CTS, then the effects are due to interactions at a more integrated (downstream) level than merely inhibition of sodium pump activity. Although ouabain inhibits Na,K-ATPase activity in both cancerous and normal cell membranes in a similar fashion, non-cancerous MCF10A cells are more sensitive to its subsequent anti-proliferative effects (Figure 2) than are tumor cells. In addition, normal breast cells, including MCF10A, primary human mammary epithelial cells 184D, and 184D-derived 184A1 cells, also exhibit a larger decrease in viability compared to cancer cells when treated with a range of ouabain, digitoxin, or bufalin concentrations (Figure 3). Our unpublished observations demonstrate that normal cells treated with ouabain display a ‘rounding up’, indicative of cells that are undergoing apoptosis [60]. Metastatic MCF7 and MDA-MB-231 cells did not display such morphology changes after ouabain treatment. These results suggest that tumor cells have the ability to more effectively resist cell death from cardiac glycosides than normal non-cancerous breast cells. 

We have characterized CTS-induced apoptosis and cellular proliferation arrest in breast cells using several experimental approaches. The oncogene-transfected and invasive metastatic breast cells have shown resistance to the negative impacts of these cardiac glycosides that are seen in normal breast cells. In order to examine the underlying mechanisms for greater cancer cell survival, we analyzed kinase activation and p53 expression with and without CTS treatment. Aberrant expression of p53 is often observed in cancer cells in a mutated form and is associated with uncontrolled proliferation [61]. Previous reports demonstrate that MCF10A cells express the wild-type form of p53, whereas MDA-MB-231 cells express mutant p53 [62]. Our results show that treatment with ouabain caused a decrease in both mutant p53 protein expression in MDA-MB-231 and wild-type p53 in MCF10CA1 cancer cell lysates, whereas wild-type p53 in MCF10A (normal cell) lysates did not change (Figure 6). The relatively low and unchanged expression of wild-type p53 in MCF10A during CTS treatment indicates that effects on p53 are unlikely to be the mechanism responsible for ouabain-induced apoptosis. Recent evidence has found that CTS can activate Src or MAPK signaling pathways, which in turn cause a reduction in p53 protein levels by inhibiting p53 translation [16]. The p53 decrease is in direct response to kinase activation, as kinase inhibitors abolished the effect. It is possible that CTS compounds, which reduce p53 levels in tumor cells, may be beneficial in treating p53 gain-of-function cancers. 

Mechanisms of kinase regulation are of key interest as signaling pathways are correlated with increased proliferation, cancer progression, transformation, invasion, and tumorigenicity [43,63-66]. Cardiotonic steroids initiate signaling pathways, such as Src/PI3K/Akt/mTOR and Ras-MEK-ERK1/2, at nanomolar concentrations [12,13]. The Src kinase is a proto-oncogene and its enhanced activation can lead to cell proliferation, invasion, and oncogenesis. A direct interaction between Src and Na,K-ATPase has been proposed, in which ouabain binding to its α-subunit receptor causes a conformational change allowing phosphorylation of the Src Y416 residue and subsequent kinase activation [47,48]. Other reports suggest that Src is activated in response to ATP/ADP cellular levels that are altered upon ouabain inhibition of the Na,K-ATPase, and not by direct interactions with α-subunits [49]. In agreement with Weigand et al., we are unable to confirm that Na, K-ATPase α-subunits interact directly with endogenous levels of Src kinase at a detectable level (Figure 7). We have observed that inhibition of Src activity by PP2 increases adhesion in MDA-MB-231 cells, but not in normal MCF10A cells (unpublished data). Thus, Src activity contributes to MDA-MB-231 cell migration, a pivotal component in cancer progression and metastasis. However, Src inhibition by PP2 does not sensitize the cells to ouabain-induced cell death (Table 3). 

Another signaling kinase, ERK1/2, also referred to as MAPK, exhibits enhanced activation when tumor cells are treated with short-term [67] or long-term (Figure 6) ouabain. The increased activation contributes to the already elevated level of active kinases present in breast cancer cells, as Src activity ranges 4-30 fold higher than that observed in normal cells [68] and abundant ERK1/2 phosphorylation is present in 65% of tumors [69,70]. Normal MCF10A cells had comparable levels of total ERK1/2 protein compared to tumor cells, but contained very low levels of activated ERK1/2 expression under normal or CTS conditions. Cancer cells treated with staurosporine also had enhanced p-ERK expression, which has been previously seen in U937 lymphoma cells [71]. A study using human cervical cancer cells found that ERK1/2 is involved in regulatory volume decrease (RVD) following hypotonic stress and calcium rise [72]. When cells were treated with a hypotonic solution, intracellular calcium levels increased which resulted in ERK1/2 activity and subsequent Cl^-^ and K^+^ channel activation. Abolishing ERK1/2 activity via a mutant raf-1 resulted in reduced Cl^-^ channel activation and hindered cell volume regulation in response to hypotonic stress. Volume-sensitive chloride channel activation, necessary for maintaining cell volume, is up-regulated during human carcinogenesis [73]. Similar to effects of hypotonic stress, CTS treatment also results in increased intracellular Ca^2+^ [74], subsequent transient activation of ERK1/2 (Figure 6) and increased survival (Figure 3) in cancer cells. We have also observed CTS-induced ERK1/2 activation in A2780CP cells (a cisplatin-chemotherapy-resistant cervical tumor cell line), and in its parental A2780 cell line (Figure S1). In addition to resistance to the apoptotic effects of CTS and subsequent high calcium levels, we have observed cancer cells are more capable of surviving hypotonic stress in 50% diluted media than non-tumorigenic MCF10A cells (Figure S2). Cancer cells often contain anti-apoptotic proteins, enhanced survival signaling, and reduced pro-apoptotic molecules, allowing for their survival during stressful conditions, such as low oxygen, alterations to pH, and nutrient deprivation [75]. Osmotic stress resistance and cell volume regulation relies on signaling kinase activation [76]. Enhanced kinase signaling and elevated Cl^-^ channel expression in tumor cells but not in normal cells may allow cancer cells to be more resistant to CTS-induced cell swelling and apoptosis. Further investigation of the mechanisms of cell volume regulation via signaling molecules may provide insight into ways of specifically targeting tumor cells for cell death.

## Conclusions

The differences in CTS sensitivity of non-cancerous cells compared to tumor cells do not directly correlate with the degree of Na,K-ATPase inhibition. Cell growth and viability of non-cancerous cells are decreased by CTS in a time and concentration-dependent manner; whereas tumor cell growth is relatively more resistant to the apoptotic effects of CTS, perhaps due to the presence of enhanced signaling pathways. The activation of ERK1/2 in response to CTS in cancer cells suggests that the Ras-Raf-MEK-ERK pathway plays an important role in cell survival, possibly through the regulation of ion channels and cell volume after an increase in intracellular Ca^2+^ and/or by inhibiting p53 protein synthesis and downstream pro-arrest or apoptosis gene expression. Increased activation of signaling molecules in CTS-treated cancer cells suggests that upstream regulators of these molecules may play a role in determining CTS sensitivity. Treatments with CTS alone do not appear to be promising candidates as a cancer therapy as tumor cells are no more sensitive to its effects than normal cells. 

## Supporting Information

Figure S1 **pERK expression in A2780 cells with ouabain treatment.** Lysates from A2780 and A2780CP cells treated with 0, 50 nM or 500 nM ouabain for 24 hours were analyzed with western blot using anti- total ERK1/2 or anti- p-ERK antibodies. Both tumor cell lines demonstrate increased levels of p-ERK after ouabain treatment.
(TIF)

Figure S2 **Osmotic stress and cancer cell viability.** MCF10 series cell lines (MCF10A, MCF10AT, and MCF10CA1) were incubated in growth media (DMEM + 10% FBS) (dark bars) or with 50% H_2_O in growth media (light bars) for 5 hours prior to the viability assay. The most oncogenic cell line, MCF10CA1, was more resistant to osmotic stress than the less tumorigenic cell lines (MCF10A and MCF10AT). Displayed as mean ± SE, n=3. Statistical significance ** p<0.001.
(TIF)
