# Supplementary figures and images for: Human Breast Tumor Cells Are More Resistant to Cardiac Glycoside Toxicity Than Non-Tumorigenic Breast Cells
Source: PLoS One. 2013 Dec 13;8(12):e84306. doi: 10.1371/journal.pone.0084306 (PMC3862803; doi:10.1371/journal.pone.0084306)

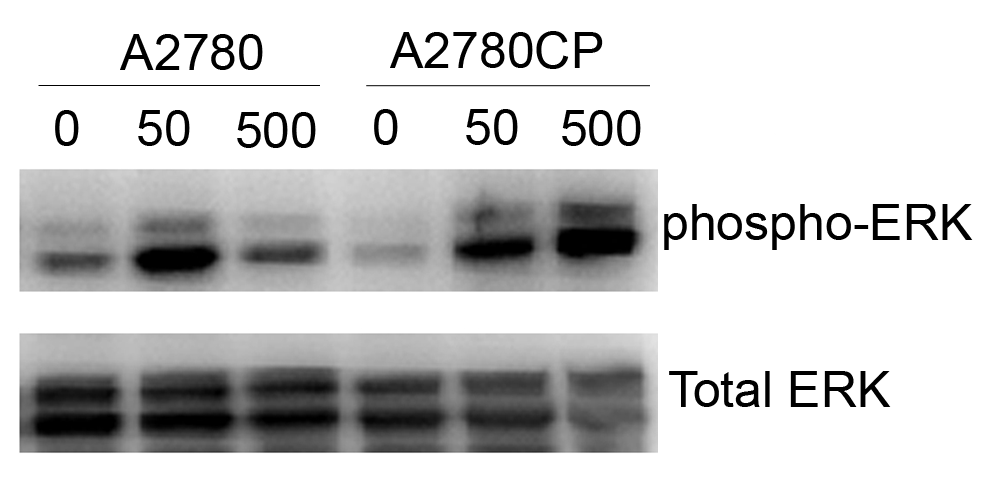

Supplement: Figure S1 — pERK expression in A2780 cells with ouabain treatment. Lysates from A2780 and A2780CP cells treated with 0, 50 nM or 500 nM ouabain for 24 hours were analyzed with western blot using anti- total ERK1/2 or anti- p-ERK antibodies. Both tumor cell lines demonstrate increased levels of p-ERK after ouabain treatment. (TIF) [file pone.0084306.s001.tif]

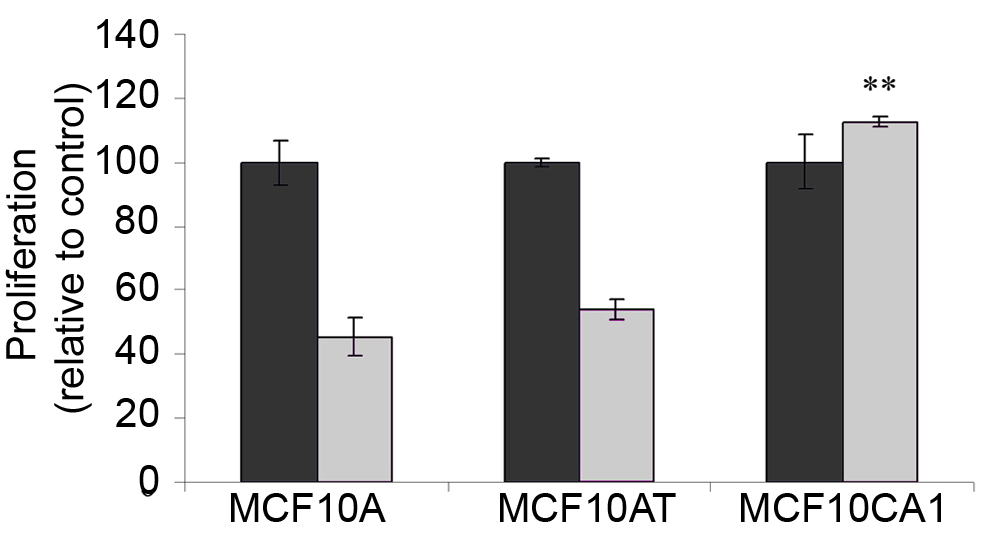

Supplement: Figure S2 — Osmotic stress and cancer cell viability. MCF10 series cell lines (MCF10A, MCF10AT, and MCF10CA1) were incubated in growth media (DMEM + 10% FBS) (dark bars) or with 50% H2O in growth media (light bars) for 5 hours prior to the viability assay. The most oncogenic cell line, MCF10CA1, was more resistant to osmotic stress than the less tumorigenic cell lines (MCF10A and MCF10AT). Displayed as mean ± SE, n=3. Statistical significance ** p<0.001. (TIF) [file pone.0084306.s002.tif]
